# Supplementary material for: Biologic Evaluation of a Heterodimeric HER2-Albumin Targeted Affibody Molecule Produced by Chemo-Enzymatic Peptide Synthesis
Source: Pharmaceutics. 2022 Nov 19;14(11):2519. doi: 10.3390/pharmaceutics14112519 (PMC9698269; doi:10.3390/pharmaceutics14112519)
Supplement: Supplementary file 1 [file pharmaceutics-14-02519-s001.zip › pharmaceutics-2007115-supplementary.pdf]

pharmaceutics

MDPI

Supplementary Materials: Biologic Evaluation of a Heterodimeric HER2-albumin Targeted Affibody Molecule Produced by Chemo-Enzymatic Peptide Synthesis

Yongsheng Liu, Rezan Güler, Yunqi Liao, Anzhelika Vorobyeva, Olof Widmark, Theodorus J. Meuleman, Anna Koijen, Leendert J. van den Bos, Robert Naasz, Vitalina Bodenko, Anna Orlova, Caroline Ekblad, Vladimir Tolmachev and Fredrik Y. Frejd

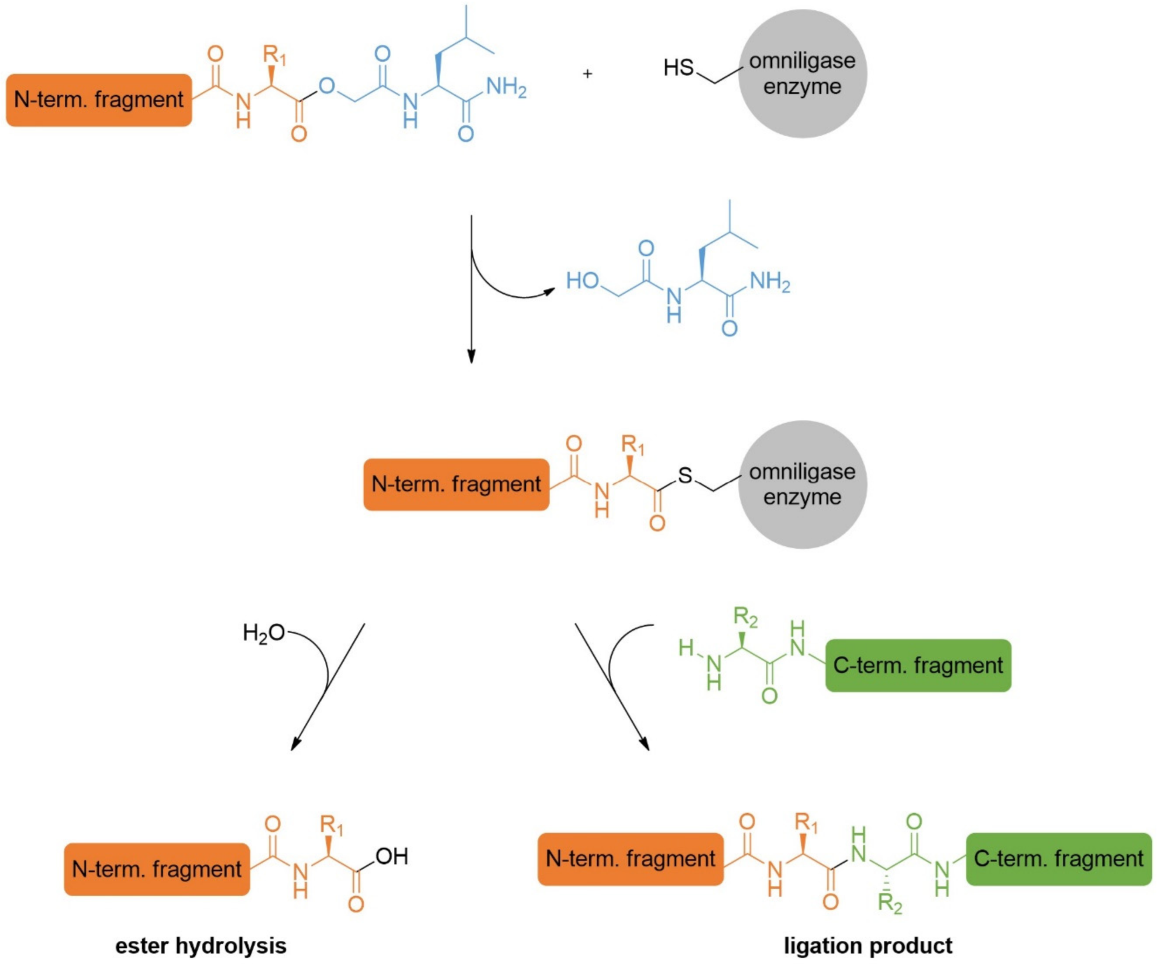

Figure S1. Reaction scheme for the Omniligase catalyzed ligation of two peptide fragments. The N-terminal fragment is modified with an ester-moiety (blue)at its C-terminus. The ligase reacts with the ester, forming a covalent thio-ester intermediate, and the organic alcohol leaves the active site. Subsequently a peptide bond is formed by nucleophilic displacement with the backbone amine of the C-terminal fragment. A possible side reaction is hydrolysis of the thio-ester.

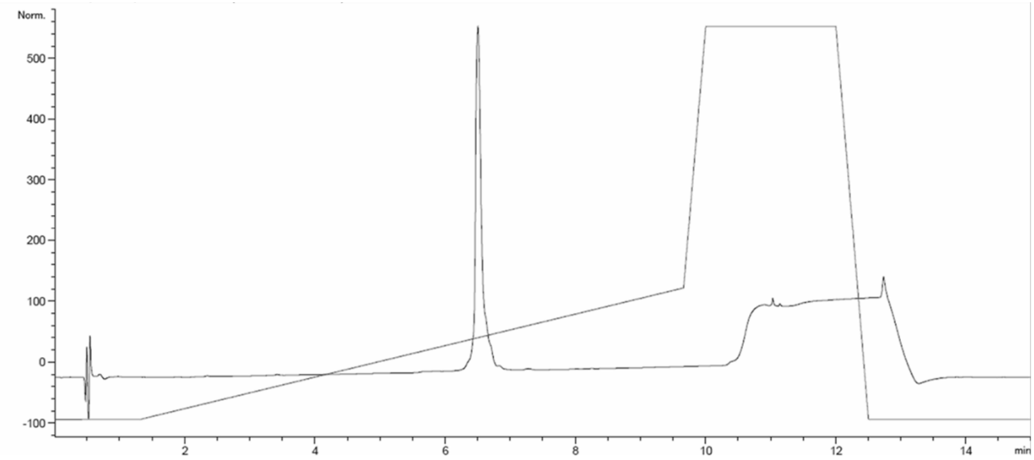

(**A**)

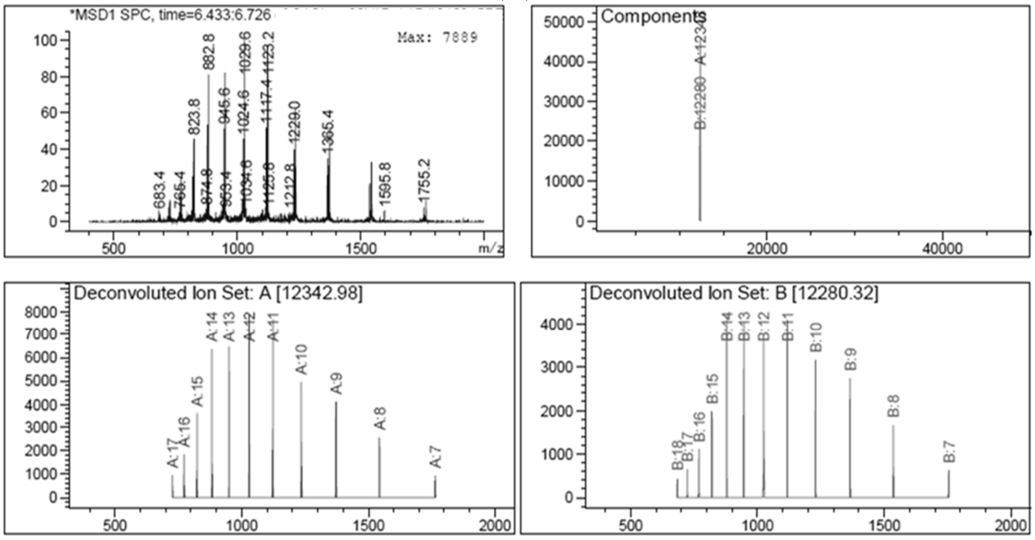

Component	Molecular Weight	Absolute Abundance	Relative Abundance
A	12342.98	46017	100.00
B	12280.32	26610	57.83

(**B**)

Figure S2. RP-UPLC-MS analysis of PEP40233 (A) Chromatogram showing the 220 nm signal and the solvent B (acetonitrile in 0.1% TFA; gradient 25-50% in 8.3 min) ratio analyzed on an Agilent Zorbax 300 SB-C8 RRHD 1.8 μm, 2.1 x 100 mm column at flow rate 0.5 mL/min at 40 °C. (B) Deconvolution of the MS TIC signal. The identity of PEP40233 (calculated Mw = 12278.9 Da) was confirmed, with a found mass of 12280.3 Da as component B and with a +63 Da Na^+ and Ka^+ adduct as component A.

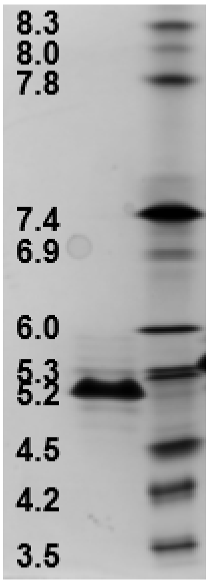

Figure S3. Isoelectric focusing of PEP40233 showing a determined pI of 5.2.

Table S1. Biodistribution of [^{177}Lu]Lu-PEP40233 in BALB/C nu/nu mice bearing SKOV-3 and BxPC3 xenografts at 48 h p.i.. Uptake is expressed as % ID/g, which is corrected for decay and presented as average value from four mice ± SD. Data for GI tract (gastrointestinal tract) are presented as %ID per whole sample. Asterisk (*) indicate significant differences ($p < 0.05$, unpaired t-test) between uptake in mice bearing SKOV-3 and BxPC3 xenografts.

Organ	Uptake at 48 h	
	**SKOV-3**	**BxPC3**
Blood	6.1 ± 1.7	5.7 ± 0.6
Heart	3.2 ± 0.5	3.0 ± 0.1
Lung	4.5 ± 0.8	4.1 ± 0.2
Liver	5.5 ± 0.7	5.4 ± 0.4
Spleen	4.8 ± 0.4	4.5 ± 0.6
Pancreas	1.3 ± 0.2	1.26 ± 0.08
Kidney	23.2 ± 1.8 *	30.5 ± 3.4
Tumor	36.9 ± 9.6 *	11.9 ± 0.3
Muscle	1.1 ± 0.2	1.2 ± 0.2
Bone	1.7 ± 0.3	1.4 ± 0.1
GI tract	3.0 ± 0.6	2.8 ± 0.2
